# GLT8D2 is a prognostic biomarker and regulator of immune cell infiltration in gastric cancer

**DOI:** 10.3389/fimmu.2024.1370367

**Published:** 2024-05-22

**Authors:** Han Wang, Jiabin Zheng, Qingyang Ma, Junchang Zhang, Yong Li

**Affiliations:** ^1^ Guangdong Cardiovascular Institute, Guangdong Provincial People’s Hospital, Guangdong Academy of Medical Sciences, Guangzhou, China; ^2^ Department of Gastrointestinal Surgery, Department of General Surgery, Guangdong Provincial People’s Hospital (Guangdong Academy of Medical Sciences), Southern Medical University, Guangzhou, China; ^3^ Department of Gastrointestinal Surgery, First Affiliated Hospital of Jinan University, Guangzhou, Guangdong, China

**Keywords:** gastric cancer, glycosylation, prognosis, biomarker, GLT8D2

## Abstract

Because of the considerable tumor heterogeneity in gastric cancer (GC), only a limited group of patients experiences positive outcomes from immunotherapy. Herein, we aim to develop predictive models related to glycosylation genes to provide a more comprehensive understanding of immunotherapy for GC. RNA sequencing (RNA-seq) data and corresponding clinical outcomes were obtained from GEO and TCGA databases, and glycosylation-related genes were obtained from GlycoGene DataBase. We identified 48 differentially expressed glycosylation-related genes and established a prognostic model (seven prognosis genes including *GLT8D2, GALNT6, ST3GAL6, GALNT15, GBGT1, FUT2, GXYLT2*) based on these glycosylation-related genes using the results from Cox regression analysis. We found that these glycosylation-related genes revealed a robust correlation with the abundance of Tumor Infiltrating Lymphocytes (TILs), especially the GLT8D2 which is associated with many TILs. Finally, we employed immunohistochemistry and Multiplex Immunohistochemical to discover that GLT8D2 serves as a valuable prognostic biomarker in GC and is closely associated with macrophage-related markers. Collectively, we established a prognostic model based on glycosylation-related genes to provide a more comprehensive understanding of prediction for GC prognosis, and identified that GLT8D2 is closely correlated with adverse prognosis and may underscore its role in regulating immune cell infiltration in GC patients.

## 1 Introduction

According to the 2020 Global Cancer Report, GC is one of the most prevalent malignancies worldwide, ranking fourth in mortality and fifth in morbidity (1). At present, a significant number of GC patients are diagnosed at an advanced tumor stage, resulting in a poor prognosis (2). Immunotherapy has emerged as a prominent therapeutic approach for advanced GC patients and has demonstrated remarkable efficacy (3). However, the efficacy of immunotherapy is limited due to the substantial tumor heterogeneity in GC, as only a small subset of patients benefited from immunotherapy, which is potentially linked to the immune microenvironment of tumors. Therefore, identifying useful biomarkers for immune checkpoint inhibitors and developing novel immunotherapeutic strategies are urgently needed.

The development of GC is a multifaceted process influenced by various factors, including environmental stimuli, epigenetic mechanisms, and protein modifications. Glycosylation represents a prevalent form of protein modification closely intertwined with numerous tumorigenesis processes. In GC, the glycosylation landscape is dramatically altered, often as a result of dysregulation of glycosyltransferases, glycosidases, and other related enzymes. Research indicates that overexpression of GnT-V induces mislocalization of E-cadherin within GC cells, consequently compromising its functionality (4, 5). In contrast, GnT-III can counteract the activity of GnT-V by regulating the glycosylation modification of E-cadherin (4). GALNT10 exhibited a positive correlation with the histological type and degree of differentiation in GC (6). GALNT2 mediates O-glycosylation of EGFR, resulting in reduced EGFR phosphorylation and inhibition of the EGFR-Akt signaling pathway, thereby impeding the onset and progression of GC (7). This suggests that glycosylation plays an important role in the occurrence and development of GC.

Acknowledging the pivotal role of glycosylation in GC pathogenesis, significant endeavors have been undertaken to delineate glycosylated gene profiles and assess their efficacy as diagnostic and therapeutic biomarkers. Currently, certain glycoprotein and glycan-associated biomarkers (referred to as carbohydrate biomarkers) are employed in human cancer screening, diagnosis, and treatment, including CA19–9, carcinoembryonic antigen (CEA), CA125, AFP, and HER2 (8). Glycosylation patterns also hold significant potential in guiding personalized treatment approaches. Expression of certain glycosylation genes correlates with response to chemotherapy, targeted agents, and immunotherapy. For instance, elevated expression of the sialyltransferase ST6GalNAc1 is linked to resistance against trastuzumab in HER2-positive GC (9). Hence, elucidating the precise pathological regulatory mechanisms underlying glycosylation modifications in GC may pave the way for novel avenues in the comprehensive treatment of GC.

Glycosylation plays a role in numerous cancer-related biological processes, yet the involvement of tumor glycosylation in immune evasion is often overlooked (10–12). Aberrant tumor glycosylation can alter the way of immune system perceives tumors, thus driving immune suppression within the tumor microenvironment (13–15). Previous studies have indicated that glycosylated histones of tumor cells can interact with lectin receptors expressed by immune cells, such as Sialic Acid-Binding Immunoglobulin-like Lectins (SIGLECs) and Macrophage Galactose-Specific Lectin (MGL), to mediate immune evasion. O-glycosylation of MUC1, CD43, and CD45, as well as the glycolipids GM2 and GD2, which carry terminal N-acetylglucosamine, can interact with MGL on macrophages, leading to increased IL-10 production and the induction of effector T-cell apoptosis, driving immune suppression processes (16). On the other hand, N-glycans stabilize PD-L1 by reducing proteasomal degradation, thereby enhancing its immune inhibitory activity (17, 18). However, the function and mechanism of glycosylation in the immune evasion of GC remain unclear. Therefore, gaining insights into the interplay between glycosylation and immune cell infiltration could offer a more comprehensive perspective on the effectiveness of cancer immunotherapy.

Investigations into prognostic signatures linked to glycosylation in cancer have yielded promising results in various malignancies, including hepatocellular carcinoma, clear cell renal cell carcinoma, and pancreatic cancer (19–21). However, similar investigations in the context of GC are scarce. Previous studies have explored cancer-related prognostic signatures associated with glycosylation in GC, these investigations have primarily focused on a restricted set of pertinent genes, possibly neglecting other critical components within the immune microenvironment. Moreover, these studies have largely remained confined to bioinformatics analysis without employing pertinent experimental validation methods, resulting in a gap in our comprehension of specific glycosylation-related genes influencing the prognosis and immune status of GC. Therefore, it is imperative to systematically analyze the relationship between glycosylation and GC, and to further explore potential novel prognostic biomarkers and therapeutic targets.

In this study, we conducted a systematic profiling of expression data specific to STAD and correlated clinical outcomes sourced from both The Cancer Genome Atlas (TCGA) and GEO databases. Additionally, we identified glycosylation-related genes utilizing data extracted from the GlycoGene DataBase. Then, we evaluated the differentially expressed glycosylation-related genes between GC tissues and adjacent normal tissues, screened for signatures associated with survival, and established a prognostic model based on glycosylation-related genes to predict the prognosis of GC patients. Furthermore, we explored the prognostic value of glycosyltransferase 8 domain-containing 2 (GLT8D2) and its potential predictive role in immunotherapy efficacy via the Tumor Immune Estimation Resource (TIMER) and immunohistochemistry. This study revealed the association between glycosylation and the immune microenvironment in GC and the possible connection and mechanism by which GLT8D2 may regulate TILs. High expression of GLT8D2 promotes the proliferation and migration of GC cells, and was also shown to be associated with a worse prognosis in GC patients.

## 2 Methods

### 2.1 Data source

RNA sequencing (RNA-seq) data for stomach adenocarcinoma (STAD), referred to as TCGA-STAD, were obtained from the TCGA database (https://portal.gdc.cancer.gov/). Additional data, including counts and fragments per kilobase of transcript per million mapped reads (FPKMs), as well as clinical information corresponding to the respective patients, were also obtained. The RNA expression data, which included the GSE19826, GSE26899, GSE54129, GSE84433 and GSE84437 datasets and contained normal and tumor tissues, were downloaded from the GEO database (https://www.ncbi.nlm.nih.gov/geo). To ensure data standardization, all the information was subjected to quantile normalization and transformed into a log2 scale. When multiple probes were used to detect a single gene symbol, the mean expression levels were calculated for analysis. Therefore, a total of 170 glycosylation-related genes obtained from the GlycoGene DataBase (GGDB; https://acgg.asia/ggdb2/) were selected as candidate genes. This study adhered to the publication guidelines stipulated by the GEO and TCGA databases.

### 2.2 Differentially expressed glycosylation-related genes

The identification of differentially expressed genes (DEGs) between tumor and adjacent normal tissues was conducted using the GEO datasets GSE19826, GSE26899 and GSE54129. This analysis was performed within the RStudio environment (version 1.2.5001) using the “limma” package, applying the following cutoff criteria for adjustment: p value < 0.05 and |log2FC| ≥ 1. Subsequently, the “heatmap” package was used to visualize the magnitude of differences across the three datasets. A Venn diagram was subsequently drawn from the selected glycosylation-related genes to determine the intersection between the candidate genes and the DEGs.

Afterwards, functional analysis was performed using the Metascape Online platform (https://Metascape.org/gp/index.html#/main/step1) (22). The differentially expressed glycosylation-related genes were input into Metascape for comprehensive functional analysis, including the construction of a protein−protein interaction (PPI) network. We applied the MCODE algorithm to identify densely connected regions within the network. A significance threshold of p < 0.05 was used for this analysis. Furthermore, functional enrichment analysis was also conducted to assess the biological functions of the differentially expressed glycosylation-related genes using Gene Ontology (GO) and Kyoto Encyclopedia of Genes and Genomes (KEGG) analyses. The criteria for GO term enrichment and KEGG signaling pathway enrichment were set at FDR < 0.05. The 10 most significant GO terms and KEGG signaling pathways were subsequently visualized using the R package “ggplot2”.

### 2.3 Construction and validation of the glycosylation-related gene prognostic model

The present study utilized the TCGA-STAD and GEO datasets (GSE84433 and GSE84437, respectively) to develop a prognostic signature based on glycosylation-related genes. The TCGA-STAD cohort served as the training cohort, while the GEO datasets GSE84433 and GSE84437 were used as the validation cohort. Univariate Cox analysis of overall survival (OS) was initially conducted to identify glycosylation-related genes associated with OS, considering a p value <0.05 to indicate statistical significance. Subsequently, the optimal model relying on prognosis-related glycosylation-related genes was identified using the Least Absolute Shrinkage and Selection Operator (LASSO) penalized Cox proportional hazards regression method through the R package “glmnet”. The signature was then established using these independent prognostic genes in accordance with their respective coefficients. Patients were divided into two groups according to the median risk score: low-risk and high-risk. Survival comparisons between the low-risk and high-risk groups were conducted using Kaplan−Meier (K−M) survival curves generated with the R package “survival”.

### 2.4 Clinical relevance investigation and prognostic nomogram construction

To furnish a quantitative predictive tool for assessing survival risk in GC patients, a nomogram was developed using differentially expressed glycosylation-related genes and clinical parameters. Additionally, calibration curves were generated to compare the predictive outcomes with actual survival data, thereby evaluating the predictive accuracy of the nomograms. The construction of the nomogram and the calibration curves was accomplished using the R package “rms”.

### 2.5 Tumor immune estimation resource database

The TIMER2.0 (https://timer.cistrome.org/) is a web-based interactive platform designed for comprehensive immune infiltration analysis across various malignancies. Six advanced algorithms were used to provide a more robust assessment of TILs levels using data from the TCGA and other tumor-related datasets. In this study, we investigated the associations between GLT8D2 expression and the expression of gene markers specific to TILs, namely, CD8+ and CD4+ T cells, B cells, monocytes, natural killer (NK) cells, dendritic cells (DCs), tumor-associated macrophages (TAMs), M1 macrophages, M2 macrophages and neutrophils, using correlation modules. To visualize the expression patterns between pairs of custom genes in GC and determine the statistical significance of the correlations, Spearman’s correlation coefficients were computed, and expression dispersion maps were generated. The gene expression levels are represented as log2 RSEM values.

### 2.6 TISIDB

TISIDB (http://cis.hku.hk/TISIDB/index.php) is an online platform that integrates diverse data sources to explore the intricate interplay between tumors and the immune system. This database proves invaluable for shedding light on the interactions between tumors and immune cells, predicting responses to immunotherapy, and identifying novel targets for immunotherapeutic interventions. It is a valuable resource for advancing research and therapies in the field of cancer immunology. In this study, we harnessed the ability of TISIDB to investigate the correlation between GLT8D2 and a comprehensive set of immune components, such as 28 TILs, in the context of GC.

### 2.7 Immunohistochemistry and multiplex immunohistochemical

This study entailed the analysis of 150 paraffin-embedded GC specimens and 30 normal specimens procured from the Shanghai Outdo Biotech Company between January 2010 and December 2015. The inclusion criteria stipulated that all samples were acquired from patients with histologically confirmed gastric adenocarcinoma and validated by expert gastrointestinal pathologists. Patient records comprised comprehensive data encompassing age, sex, tumor location, TNM stage, histological grade, Lauren’s classification, treatment history, and detailed follow-up information for survival analysis. The exclusion criteria encompassed patients who had undergone chemotherapy or radiotherapy before surgery and those with synchronous or metachronous malignancies. Multiplex Immunohistochemistry (PANOVUE kit, #10234100050) was employed to assess the expression levels of GLT8D2 and CD68, aiming to establish a correlation between GLT8D2 expression and CD68 expression. Anti-GLT8D2 (1:1000 dilution; Bioss, bs-8302R) and anti-CD68 (1:2000 dilution; Cell Signaling Technology, #97778) antibodies were utilized. Immunohistochemistry was carried out according to the DAB kit of Fujian Maxim Company (DAB-0031), Anti-GLT8D2 (1:100 dilution; Bioss, bs-8302R) antibody was used. Staining intensities were classified into four categories: negative (-), weak (+), moderate (++), and strong (+++).

### 2.8 Cell transfection and lentiviral infection

Gastric cancer cell line AGS, purchased from the Cell Bank of Chinese Academy of Sciences (Shanghai), was used in this study and treated with DMEM/F12 (Gibco, CAT# C11330500BT, Beijing, China) medium combined with 10% fetal bovine serum FBS (Gibco, CAT# 10099141C, Beijing, China). China). GeneChem(Shanghai, China) provided the GLT8D2-knockdown lentiviral vector. GLT8D2 was cloned into GV341 vector (GeneChem, Shanghai, China) to construct GLT8D2 lentiviral expression vector. Lentivirus transduction was generated and purified according to the manufacturer’s instructions. Puromycin (2 μg/ml) was added to screen the transgenic cells.

### 2.9 Cell viability, colony formation, and wound healing assays

A quantity ranging from 1000 to 1500 cells were evenly distributed across the wells of 96-well culture plates. Subsequently, the assessment of cell viability was carried out using a Cell Counting Kit-8 (CCK-8) (Beyotime, CAT# C0048M) following a 2-hour incubation period at 37°C. This evaluation was conducted at multiple time points, specifically 0, 24, 48, and 72 hours post-seeding, in strict adherence to the guidelines provided by the manufacturer. In colony formation assays, 500 cells were seeded per well in six-well plates for experiments, and the cells were cultured for two weeks. Subsequently, the colonies were fixed with 4% paraformaldehyde for 15 minutes and stained with crystal violet (Beyotime Biotechnology, CAT# C0121) for 15 minutes. In the wound healing assays, we used cell culture dishes to create a defined wound and observed the migration capability of the cells during the healing process. At specific time intervals (0 h, 12 h, and 24 h), we documented and measured the extent of wound closure to assess the cell migration and healing ability.

### 2.10 Cell migration assays

Migration assays were carried out using transwell plates with 8-μm pores. In the migration assay, cells were placed in the top compartment with 0.2 ml of serum-free medium, while 0.8 ml of culture media supplemented with 10% fetal bovine serum was added to the bottom chamber. After the cells were incubated for 24 hours, they were fixed with 4% paraformaldehyde for 15 minutes and subsequently stained with crystal violet for 15 minutes. Unmigrated cells were then removed from the top layer using cotton swabs. Migrating cells were observed and imaged using a 10× microscope (Olympus CKX53).

### 2.11 Statistical analysis

Statistical analyses were performed using the Statistical Package for the Social Sciences (SPSS, version 26.0) and GraphPad Prism (version 8.0). K−M plots were generated to construct survival curves. In these KM plots, as well as in the analysis conducted using the TIMER2.0 and TISIDB tools, hazard ratios (HRs) and p values were computed using the log-rank test. Spearman’s correlation coefficient was utilized to evaluate the correlation between GLT8D2 expression and immune infiltration. Univariate and multivariate Cox regression analyses were executed with the R package “survival”, providing HRs along with their corresponding 95% confidence intervals (CIs). Additionally, the differences among various clinical factors were evaluated using independent t tests, with statistical significance denoted by a p value < 0.05.

## 3 Results

### 3.1 Differentially expressed glycosylation-related gene signatures in GC

**Figure 1:**
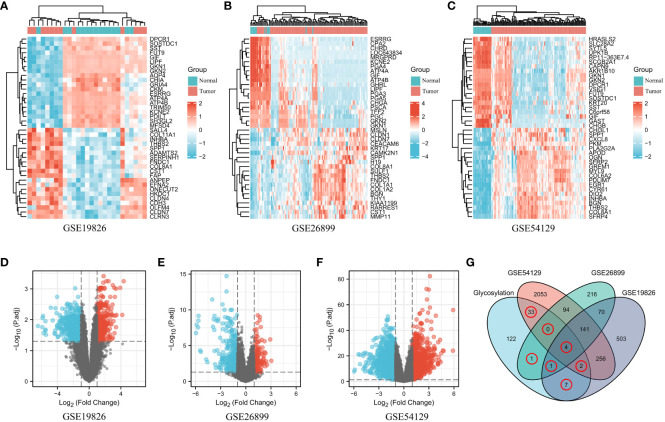
Differentially expressed glycosylation-related gene signatures in GC. **(A–C)**. The expression patterns of glycosylation-related genes in both normal and tumor samples were examined across the GEO datasets: GSE19826 **(A)**, GSE26899 **(B)**, and GSE54129 **(C)**. Genes were categorized based on their expression levels, with high expression represented by the color red and low expression represented by the color blue. **(D–F)**. Volcano plots showing the dysregulated glycosylation-related genes in the three aforementioned GEO datasets. **(G)** Venn diagram showing the dysregulated glycosylation-related genes common to the four datasets.

The GEO datasets used in this study are provided in **Supplementary Table S1**. After conducting the differential gene analysis, a total of 984 dysregulated genes were identified from the GEO dataset GSE19826, with 338 genes exhibiting upregulation and 646 genes exhibiting downregulation (**Figure 1A**). Additionally, from the GEO dataset GSE26899, 527 dysregulated genes were found, consisting of 174 upregulated genes and 353 downregulated genes (**Figure 1B**). Finally, the GEO dataset GSE54129 yielded 2,583 dysregulated genes, of which 1,134 genes were upregulated and 1449 genes were downregulated (**Figure 1C**). Furthermore, the dysregulated genes in the three aforementioned datasets were visualized in a more intuitive manner using volcano plots (**Figures 1D–F**). The glycosylation genes obtained from the GlycoGene DataBase were experimentally validated and are listed in **Supplementary Table S2**. To obtain the “differentially expressed glycosylation-related genes”, the differentially expressed genes (DEGs) from the GEO datasets were compared with the glycosylation-related gene set using a Venn diagram, which revealed 48 intersecting glycosylation-related genes among the four datasets (**Figure 1G**).

**Figure 2:**
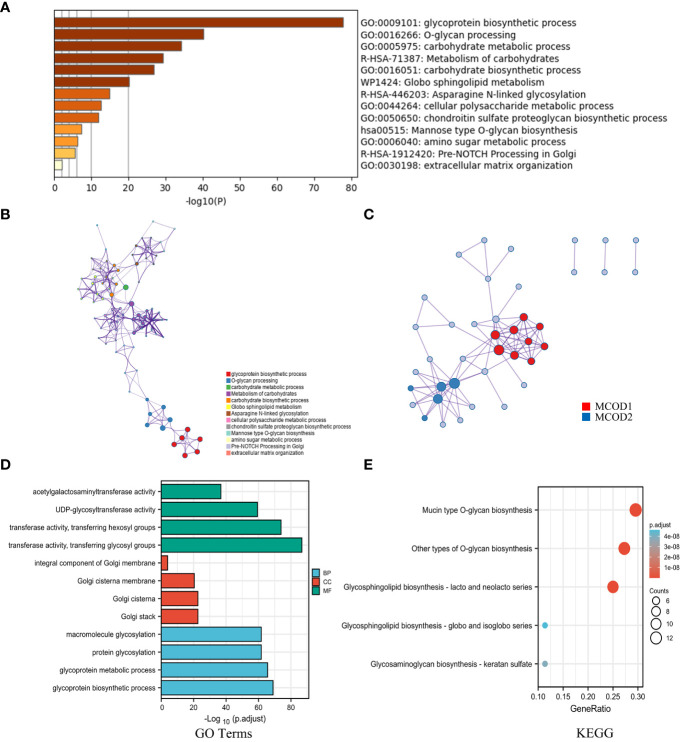
The mechanisms underlying glycosylation signatures in GC. **(A)** Bar plot showing the distribution and relationships of the different functions according to the GO and KEGG analyses based on Metascape Online. **(B)** Network showing the distribution and relationships of the different functions according to the GO and KEGG analyses based on Metascape Online. **(C)** PPI network and MCODE showing the hub genes among the glycosylation-related genes. **(D)** GO enrichment analysis; BP, biological process; CC, cellular component; MF, molecular function. **(E)** KEGG pathway annotation.

To investigate the mechanisms underlying glycosylation signatures in GC, a comprehensive functional analysis was conducted using Metascape Online. Our findings indicated that the dysregulated glycosylation genes are primarily associated with various biological processes, such as the response to glycoprotein biosynthetic process, O-glycan processing, carbohydrate metabolic process, and metabolism of carbohydrates, as revealed by Gene Ontology (GO) analysis (**Figure 2A**). Moreover, KEGG pathway analysis also demonstrated that these dysregulated glycosylation genes were significantly enriched in pathways related to glycoprotein biosynthetic process, O-glycan and N-linked glycosylation, and cellular polysaccharide metabolic process (**Figure 2B**). These results prompted us to explore the correlation between the glycosylation gene set and the progression of GC. Furthermore, through the utilization of the protein−protein interaction (PPI) network and the MCODE plugin in Metascape Online, we identified significant modules within these glycosyltransferase genes (**Figure 2C**). Module 1 included *FUT2, FUT3, FUT4, FUT9, GCNT2, B4GALT1, B4GALT4, B3GNT3, and ST3GAL6*. Module 2 includes *GCNT1, GALNT7, GALNT12, B3GNT6, and ST6GALNAC1*. By utilizing the TCGA database, we found that the most enriched terms in terms of biological process (BP), cellular component (CC), and molecular function (MF) were “transferase activity”, “Golgi stack”, and “glycoprotein biosynthetic process”, respectively (**Figure 2D**). Moreover, functional enrichment analysis revealed that the signaling pathway most relevant to the glycosyltransferase genes was O-glycan biosynthesis (**Figure 2E**).

### 3.2 The establishment and verification of a glycosylation-related prognostic model

**Figure 3:**
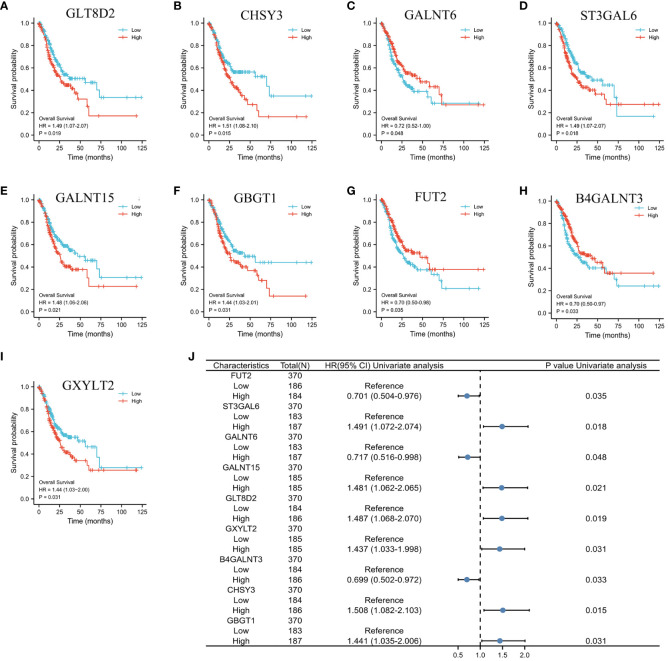
K−M plots and forest plot of glycosylation-related prognostic factors. **(A–I)** Kaplan−Meier plots showing the glycosylation-related genes with prognostic value. **(J)** Forest plot showing the results of the univariate Cox regression analyses.

Initially, the genes significantly associated with prognosis were detected through the application of univariate Cox regression analysis. As depicted in **Figures 3A–I**, nine glycosylation-related genes were identified as prognostic genes: *GLT8D2, CHSY3, GALNT6, ST3GAL6, GALNT15, GBGT1, FUT2, B4GALNT3, and GXYLT2*. **Figure 3J** displays a forest plot presenting the outcomes of the univariate Cox regression analysis. Therefore, a prognostic model was established based on the Cox regression coefficient as follows: risk score= [expression level of FUT2 × (-0.11)] + [expression level of ST3GAL6 × 0.26] + [expression level of GALNT6 × (-0.03)] + [expression level of GALNT15 × 0.01] + [expression level of GLT8D2 × 0.14] + [expression level of GXYLT2 × 0.01] + [expression level of GBGT1 × (-0.05)].

### 3.3 The predictive model construction for GC patients

**Figure 4:**
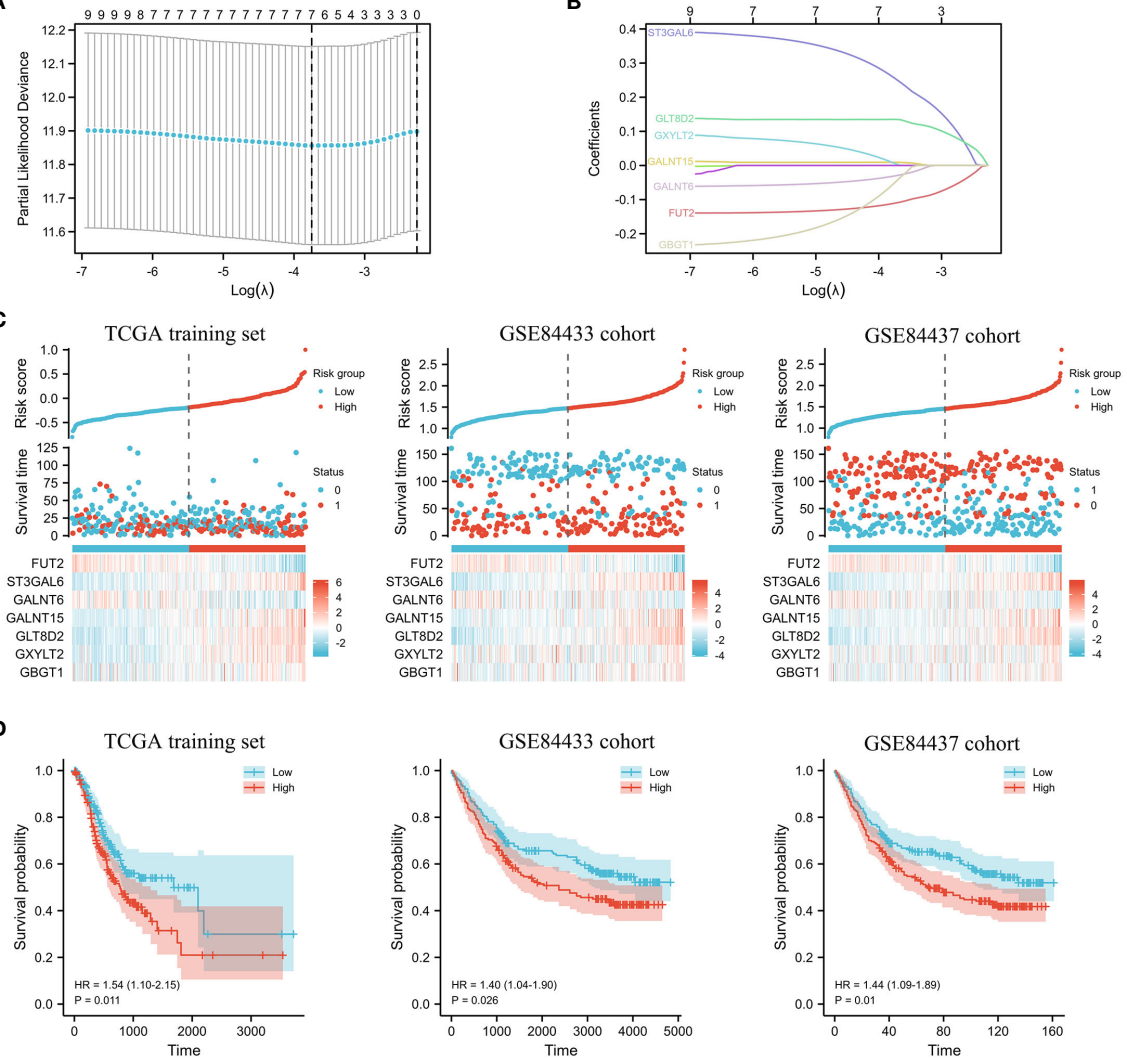
The predictive model constructed for GC patients. **(A)** Partial likelihood deviance of DEGs. **(B)** LASSO regression and coefficient values of DEGs. **(C)** Risk score distribution, survival status, and expression of 7 DEGs for GC patients in the low- and high-risk groups in the TCGA training set and the GSE84433 and GSE84437 cohorts. **(D)** KM survival analyses of 7 DEGs for GC patients in the low- and high-risk groups in the TCGA training set and the GSE84433 and GSE84437 cohorts.

In the training cohort, LASSO regression was adopted to analyze the data according to the univariate analysis procedure described above (**Figure 4A**). After conducting calculations that involved combining the coefficients from the LASSO analysis with the levels of gene expression, we identified a set of seven prognostic genes: *GLT8D2, GALNT6, ST3GAL6, GALNT15, GBGT1, FUT2, and GXYLT2* (**Figure 4B**). Employing these seven genes, we computed an individualized risk score for each patient, and the threshold for distinguishing between the high-risk and low-risk categories was established at the median value (**Figure 4C**). This study revealed that OS was significantly worse in high-risk patients than in low-risk patients in the TCGA training set (**Figure 4D**, P<0.05). Similar results were obtained in the validation sets GSE84433 and GSE84437 (**Figure 4D**, P<0.05).

### 3.4 Construction of the nomogram

**Figure 5:**
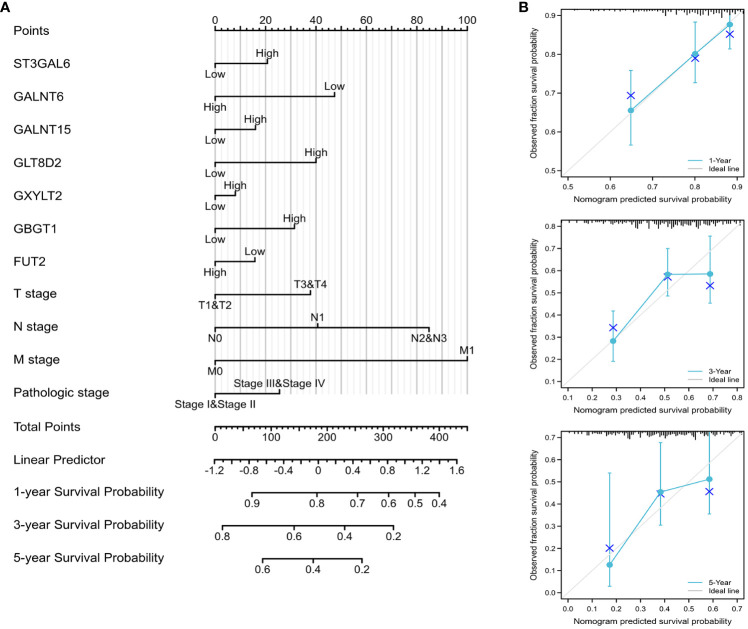
Construction of the prognostic nomogram **(A)** Nomogram designed to predict 1-, 3-, and 5-year OS in the complete TCGA cohort. **(B)** Calibration curves of the nomogram assessing the agreement between projected and observed 1-, 3-, and 5-year survival rates across the entire TCGA cohort. A dashed line at 45° indicates a flawless prediction, with the actual performance of our nomogram depicted by the blue lines.

A predictive glycosylation-related prognostic nomogram was established via multivariate analysis. Therefore, using seven prognostic genes and certain clinicopathological factors, we developed a prognostic nomogram that serves as a valuable quantitative tool for predicting the survival prospects of individual patients (**Figure 5A**). Furthermore, the predictive accuracy for overall survival was evaluated via calibration curves. Importantly, the calibration curves of this prognostic nomogram demonstrated excellent agreement between the predicted and actual survival rates at the 1-, 3-, and 5-year milestones across the entire TCGA cohort (**Figure 5B**).

### 3.5 Correlation between immune infiltration and GLT8D2 expression in GC

**Figure 6:**
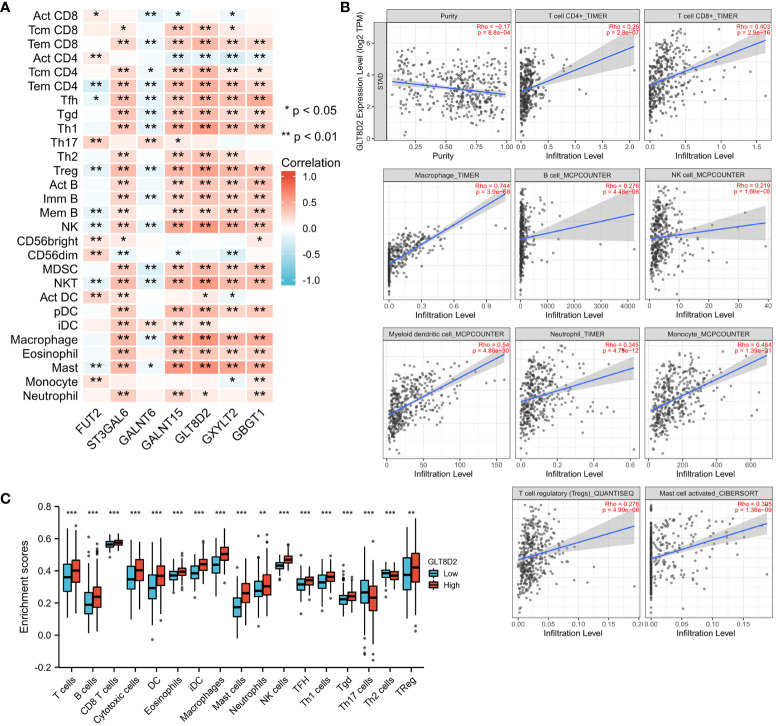
Correlations between immune infiltration and glycosylation-related genes in GC. **(A)** Correlation matrix of seven glycosylation-related genes and the abundance of TILs. The red dots represent a positive correlation, and the blue dots represent a negative correlation. **(B)** Association between GLT8D2 expression and immune cell infiltration in STAD according to TIMER data. **(C)** Differences in immune cells between patients with high or low GLT8D2 expression in tumors in the TCGA database. *, **, and *** represent P<0.05, P<0.01, and P<0.001 respectively.

Immune cell infiltration is crucial in tumor progression. Therefore, to further explore the association of glycosylation-related genes with immunity, correlation analysis was conducted between seven glycosylation-related genes and immune functions via the TISIDB platform. As shown in **Figure 6A**, these glycosylation-related genes were strongly correlated with the abundance of TILs, especially GLT8D2, which is associated with many TILs.

As such, we utilized the TIMER platform to evaluate the association between GLT8D2 expression and immune cell infiltration in STAD. GLT8D2 expression was adversely correlated with the purity of the STAD cells (rho = -0.17, p < 0.00088). Our findings revealed a robust correlation between GLT8D2 and TILs. Specifically, a high level of GLT8D2 expression was positively associated with the degree of infiltration by various immune cell populations, including macrophages (rho = 0.744), CD8^+^ T cells (rho = 0.403), CD4^+^ T cells (rho = 0.26), B cells (rho = 0.276), monocytes (rho = 0.464), neutrophils (rho = 0.345), T-cell regulatory cells (rho = 0.276), NK cells (rho = 0.219), and myeloid dendritic cells (rho = 0.54) (**Figure 6B**). Importantly, all p values were markedly less than 0.001. The TCGA database was also used to assess the difference in immune cells between patients with high- or low-grade GLT8D2 tumors. Similar results were obtained (**Figure 6C**). These findings collectively underscore the pivotal role of GLT8D2 in orchestrating immune infiltration within the context of GC.

### 3.6 GLT8D2 expression is correlated with macrophage-related marker expression and poor prognosis in GC patients

**Figure 7:**
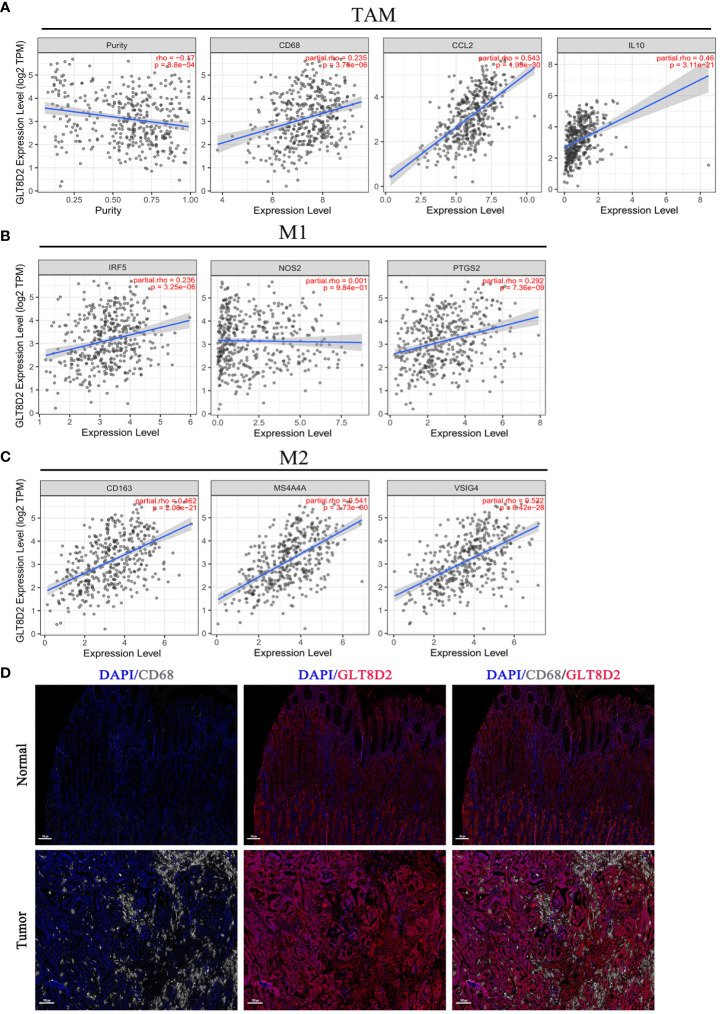
GLT8D2 expression is correlated with macrophage-related marker expression and poor prognosis in GC patients. **(A–C)** Associations between GLT8D2 expression and macrophage-related markers, including tumor-associated macrophages (TAMs), M1-type macrophages, and M2-type macrophages, in STAD according to TIMER analysis. **(D)** Multiplex immunohistochemical staining showing the correlation between GLT8D2 (red) and CD68 (white) expression.

**Figure 8:**
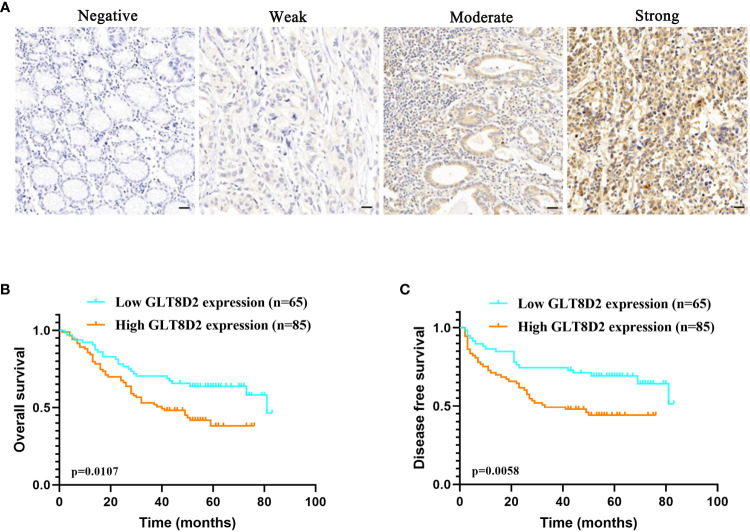
GLT8D2 serves as a valuable prognostic biomarker in GC. **(A)** Immunohistochemistry image showing GLT8D2 expression based on the immunohistochemical score. Patients were classified into four groups: negative (–), weak (+), moderate (++) and strong (+++) staining. **(B–C)** Relationships between GLT8D2 expression and overall survival (OS) **(B)** or disease-free survival (DFS) **(C)** outcomes in GC patients.

Evidently, GLT8D2 exhibited a significant correlation with a majority of the marker sets associated with tumor-associated macrophages (TAMs), M1-type macrophages, and M2-type macrophages in STAD. Specifically, this study revealed strong correlations between GLT8D2 and TAM markers, including CD68, chemokine ligand (CCL)-2 and Interleukin 10 (IL10), in STAD. Additionally, GLT8D2 displayed robust correlations with M1 phenotype markers, such as Interferon Regulatory Factor 5 (IRF5) and Prostaglandin-Endoperoxide Synthase 2 (PTGS2), as well as with M2 phenotype markers, including CD163, V-Set and Immunoglobulin Domain Containing 4 (VSIG4), and Membrane Spanning 4-Domains A4A (MS4A4A) (**Figures 7A–C**). All p values were markedly less than 0.001. Moreover, we employed a multiplex immunohistochemical approach to assess the correlation between GLT8D2 and CD68 expression. Our findings demonstrated that elevated GLT8D2 expression was associated with increased CD68 infiltration (**Figure 7D**). Concurrently, we investigated the relationship between GLT8D2 expression and clinicopathological characteristics in GC patients via immunohistochemistry (IHC) (**Table 1**). By scoring the staining intensity, we classified the expression levels of GLT8D2 into four groups: negative (–), weak (+), moderate (++) and strong (+++) staining (**Figure 8A**). The results of this study indicated that high GLT8D2 expression was correlated with poorer OS and disease-free survival (DFS) in GC patients (**Figures 8B, C**). Furthermore, univariate and multivariate Cox proportional hazards regression analyses of OS and DFS in GC patients revealed that GLT8D2 was an independent prognostic risk factor (**Tables 2**, **3**). Consequently, our findings support the assertion that GLT8D2 is a valuable prognostic biomarker in GC and is closely associated with immune infiltration.

**Table 1:** Associations of GLT8D2 expression with clinical parameters in GC patients.

Characteristic	GLT8D2		
	Low (%)	High (%)	*P*
Age (years)			0.077
<60	37(50.7)	36(49.3)	
≥60	28(36.4)	49(63.6)	
Gender			0.848
Male	40(44.0)	51(56.0)	
Female	25(42.4)	34(57.6)	
Tumor size			0.174
≤5 cm	41(47.7)	45(52.3)	
>5 cm	23(36.5)	40(63.5)	
Borrmann type			0.981
I-II	11(42.3)	15(57.7)	
III-IV	37(42.0)	61(58.0)	
Differentiation			**0.004**
Well+ moderate	22(64.7)	12(35.3)	
poor	34(35.8)	61(64.2)	
pTNM stage			**0.030**
I-II	32(54.2)	27(45.8)	
III-IV	33(36.3)	58(63.7)	
Depth of invasion			**0.023**
T1/2	20(60.6)	13(39.4)	
T3/4	45(38.5)	72(61.5)	
Lymph node metastasis			0.13
N0	26(52.0)	24(48.0)	
N+	39(30.0)	61(70.0)	
Distant metastasis			0.272
M0	61(45.2)	74(54.8)	
M1	4(26.7)	11(73.3)	
CEA level (μg/L)			0.155
≤5	57(46.0)	67(54.0)	
>5	8(30.8)	18(69.2)	
LVI			**0.027**
Yes	10(27.0)	27(73.0)	
No	48(48.0)	52(52.0)	
PNI			**0.046**
Yes	3(18.8)	13(81.3)	
No	53(44.9)	65(55.1)	

Bold values indicate P < 0.05.

**Table 2:** Univariate and multivariate analyses for OS in GC patients.

Variable	Univariate			Multivariate		
	HR	95%CI	*P*	HR	95%CI	*P*
Age (years)						
≥60 vs.<60	0.633	0.346–1.155	0.136			
Gender						
Male vs. Female	1.921	1.079–1.3.419	**0.026**	1.946	1.127–3.361	**0.017**
Tumor size						
>5 cm vs. ≤5 cm	1.774	0.958–3.284	0.068	1.866	1.060–3.285	**0.031**
Borrmann type						
III-IV vs. I-II	3.327	1.107–9.998	**0.032**	3.999	1.344–11.89	**0.013**
Differentiation						
poor vs. Well+ moderate	0.5922	0.27–1.297	0.190			
Depth of invasion						
T3–4 vs. T1–2	4.368	0.568–33.594	0.157			
Lymph node metastasis						
N+ vs. N0	4.050	1.632–10.048	**0.003**	4.815	2.05–11.306	**0.001**
CEA level (μg/L)						
>5 vs. ≤5	1.052	0.498–2.223	0.895			
LVI						
Present vs. none	1.291	0.680–2.453	0.435			
PNI						
Present vs. none	1.047	0.372–2.944	0.931			
GLT8D2						
High vs. Low	2.165	1.149–4.078	**0.017**	2.078	1.155–3.738	**0.015**

Bold values indicate P < 0.05.

**Table 3:** Univariate and multivariate analyses for DFS in GC patients.

Variable	Univariate			Multivariate		
	HR	95%CI	*P*	HR	95%CI	*P*
Age (years)						
≥60 vs.<60	0.672	0.367–1.231	0.672			
Gender						
Male vs. Female	1.675	0.950–2.952	0.074	1.752	1.015–3.023	**0.044**
Tumor size						
>5 cm vs. ≤5 cm	1.832	0.981–3.421	0.058	2.008	1.133–3.557	**0.017**
Borrmann type						
III-IV vs. I-II	3.332	1.114–9.962	**0.031**	3.976	1.339–11.81	**0.013**
Differentiation						
Poor vs. Well+ moderate	0.622	0.287–1.347	0.229			
Depth of invasion						
T3–4 vs. T1–2	4.532	0.591–34.738	0.146			
Lymph node metastasis						
N+ vs. N0	3.981	1.608–9.855	**0.003**	5.062	2.157–11.88	**0.001**
CEA level (μg/L)						
>5 vs. ≤5	0.961	0.459–2.012	0.915			
LVI						
Present vs. none	1.708	0.906–3.222	0.098			
PNI						
Present vs. none	0.885	0.317–2.475	0.836			
GLT8D2						
High vs. Low	2.142	1.149–3.993	**0.017**	2.091	1.167–3.748	**0.013**

Bold values indicate P < 0.05.

### 3.7 GLT8D2 knockdown blocks the proliferation and metastasis of GC cells *in vitro*

**Figure 9:**
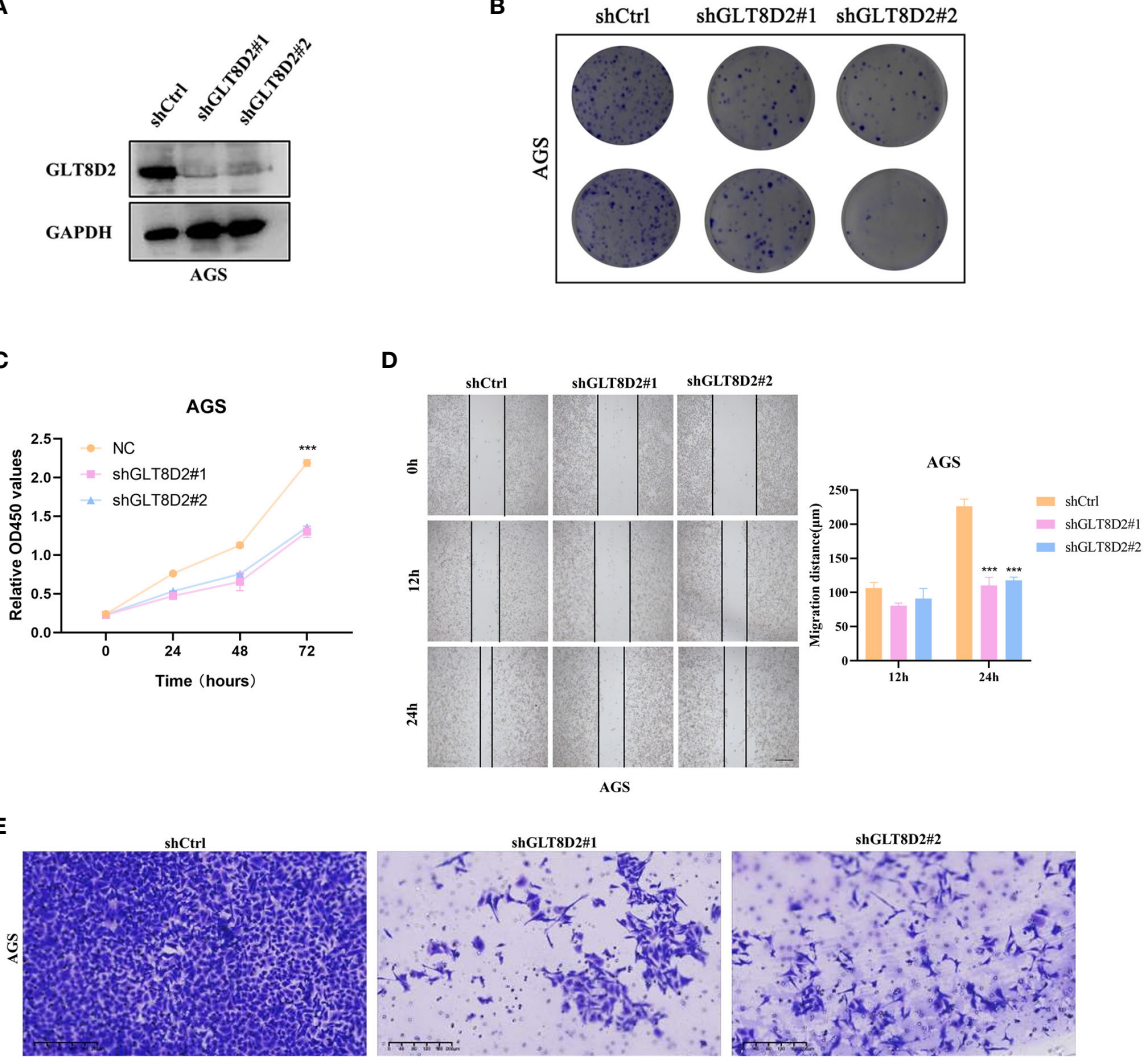
GLT8D2 knockdown blocks the proliferation and metastasis of GC cells *in vitro*. **(A)** Evaluation of the efficiency of shRNA via Western blotting. **(B)** Colony formation assays showing the clonogenic capacity of GC cells following GLT8D2 knockdown. **(C)** Cells growth ability after GLT8D2 knockdown were determined by CCK8 assay. **(D)** The wound healing assay showing the migration ability of GC cells following GLT8D2 knockdown. **(E)** Transwell assay showing the migratory capacity of GC cells following GLT8D2 knockdown.

To gain a deeper understanding of the impact of GLT8D2 in GC, we explored phenotypic alterations in GC cells following GLT8D2 knockdown. The effectiveness of GLT8D2 knockdown was verified by western blotting (**Figure 9A**). Colony formation assays and cell viability demonstrated a reduction in the clonogenic capacity of GC cells following GLT8D2 knockdown (**Figures 9B, C**). Furthermore, the wound healing assay revealed wider wounds after the same 24-hour interval in the GLT8D2 deficiency groups than in the shCtrl group (**Figure 9D**). In addition, GLT8D2 knockdown significantly diminished the migratory ability of GC cells, as evidenced by cell migration assays (**Figure 9E**). Collectively, these findings indicate a pivotal role for GLT8D2 in the proliferation and migration of GC cells.

## 4 Discussion

Increasing evidence indicates that glycosylation plays a pivotal role in tumorigenesis and the efficacy of cancer treatments. In the present study, we focused on glycosylation-related genes and investigated their impact on the prognosis of GC patients. Our objective was to elucidate glycosylation-related prognostic models and their relationship with the GC immune microenvironment, aiming to further identify potential biomarkers for prognosis assessment and targeted therapy. A comprehensive bioinformatics study was subsequently performed to systematically analyze glycosylation-related genes associated with poor prognosis in GC patients, and a glycosylation-based prognostic model was established by using the GEO, TCGA, and GlycoGene databases. Additionally, we showed that high expression of GLT8D2 was associated with poor prognosis in GC patients and revealed novel insights into the key role of GLT8D2, which may serve as a prognostic biomarker associated with immune infiltration in GC.

Glycosylation plays a role in numerous cancer-related biological processes, including inflammation, immune surveillance, cell−cell adhesion (4, 5), cell-matrix interactions (23), intercellular and intracellular signal transduction (24–27), and cellular metabolism (28, 29). Tumor classification studies based on glycosylation-related gene expression profiles have begun to emerge (30–32). Subtypes of colorectal cancer patients with poor prognoses have been identified using glycan gene markers, among which the loss of GALNT6 gene expression has been associated with cancer cell invasion and chemoresistance and has been highlighted as a prognostic biomarker (33). Research has indicated that the overexpression of GnT-V results in the mislocalization of E-cadherin within GC cells, leading to functional impairment. The primary mechanism involves the addition of N-glycan chains with β-1,6-GlcNAc branches mediated by GnT-V to E-cadherin, promoting incorrect assembly and ineffective adhesive connections, thereby affecting cell−cell adhesion and subsequently contributing to tumor metastasis (4, 5). Therefore, exploring the biological significance of glycosylation in GC is advantageous for deciphering the pathological regulatory mechanisms involved in cancer biology, which may help in identifying novel biomarkers for prognosis and targeted therapy.

In this study, we analyzed DEGs from the GEO datasets GSE19826, GSE26899, and GSE75241 and intersected them with a glycosylation-related gene set obtained from the GlycoGene DataBase, resulting in the identification of 48 glycosylation-related genes. Subsequently, functional analysis of these 48 glycosylation-related genes revealed that these genes were associated with various biological processes, including the response to glycoprotein biosynthetic processes and O-glycan processing. We also employed univariate Cox and multivariateCox regression analyses to identify 9 out of 48 adverseprognosis-associated glycosylation-related genes and establish a glycosylation-based prognostic model. Among these proteins, GALNT6 has been reported to promote the occurrence of breast cancer through abnormal glycosylation of the mucin protein MUC1 (34). In addition, previous research has revealed that the hypermethylation of ST3GAL6 is strongly correlated with Epstein–Barr virus-associated gastric carcinomas (35). In addition, GXYLT2 has also been reported to be a potential diagnostic and prognostic biomarker for GC by bioinformatics analysis (36). This study discovered that GLT8D2 is highly expressed in GC and is closely associated with poor prognosis by bioinformatics analysis and clinical samples. Cellular functional studies also suggested that GLT8D2 affects the proliferation and migration of GC cells. As a glycosyltransferase, GLT8D2 may modify the substrate protein by glycosylation, thereby affecting its stability, localization, interaction and activity, and then regulate the occurrence and development of tumors (24–27). Moreover, glycosylation is essential for the function of adhesion molecules such as integrins and cadherins on the cell membrane (27). Abnormal expression of GLT8D2 may affect the glycan modification of these molecules, reduce the adhesion of cells to the extracellular matrix, enhance cell migration and invasion, and promote tumor spread and growth. These studies suggest that glycosylation-related genes especially GLT8D2 may play a crucial role in the development and progression of GC. Therefore, we believe that our study may contribute to providing new insights into GC treatment.

In the cancer microenvironment, TILs have been demonstrated to play a crucial role in the initiation and progression of cancer (37–39). They may show the characteristics of promoting or inhibiting tumor growth in different types of cancer and different stages of the same type of cancer (40, 41). For CD8+ CTLs (cytotoxic T lymphocytes), they act as tumor suppressors by triggering a cytolytic reaction by recognizing tumor-specific antigens presented by the major histocompatibility complex (MHC) (42). In addition, regulatory T cells (Tregs) and myeloid-derived suppressor cells (MDSCs) can create an immunosuppressive tumor microenvironment by secreting inhibitory cytokines (such as IL-10 and TGF-β), depleting trophic factors, and directly inhibiting effector T cell function, thereby promoting tumor progression (43). Consequently, we observed a robust correlation between glycosylation-related genes and immune-infiltrating cells. To ensure the depth and practical applicability of our research, it is imperative to focus on the comprehensive exploration of the most promising or scientifically significant genes in subsequent studies. Subsequently, our analysis revealed that the GLT8D2 gene, a novel glycosyltransferase situated on chromosomal region 12q23.3, exhibited noteworthy correlations and biological significance across multiple dimensions. Previous research revealed that GLT8D2 is involved in the pathogenesis of nonalcoholic fatty liver disease (NAFLD) by negatively regulating microsomal triglyceride transfer protein (MTP) in HepG2 cells (44), and the GLT8D2/FGFR/PI3K/AKT signaling axis was found to be a significant contributor to platinum-based chemotherapy resistance in ovarian cancer (45). However, the biological functions and association with immune infiltration of GLT8D2 in GC remain unclear.

Thus, we systematically investigated the association between GLT8D2 expression and the degree of immune infiltration in GC. Our study revealed strong correlations between GLT8D2 expression and TILs, including CD8^+^ T cells, CD4^+^ T cells, Treg cells, B cells, neutrophils, dendritic cells (DCs), natural killer (NK) cells, and monocytes, particularly macrophages. Macrophages are a distinct type of immune cell classified into M1 and M2 subtypes and play critical roles in angiogenesis (46), invasion (47), and antitumor immunity (48). Additionally, our research demonstrated the association between GLT8D2 expression and macrophage markers in GC via TIMER. Clearly, GLT8D2 expression was strongly correlated with TAM markers, including CD68, CCL-2 and IL10. Moreover, by employing an immunohistochemical approach, this study demonstrated that elevated GLT8D2 expression was associated with increased CD68 infiltration and led to poor prognosis in GC patients. This suggested that GLT8D2-regulated TILs mainly play a role in promoting tumor progression, and the mechanism may be related to the immune escape caused by galactose-specific lectin (MGL) of macrophages, which can leading to increased IL-10 production and the induction of effector T-cell apoptosis, driving immune suppression processes (16). These findings discovered that GLT8D2 may potentially regulate TAM polarization, which could enhance the effectiveness of immunotherapy by targeting GLT8D2. High expression of GLT8D2 was also shown to be associated with a worse prognosis in GC patients. Taken together, these findings indicate that GLT8D2 plays a significant role in recruiting and modulating TILs in GC. Further investigations into the molecular mechanisms and functions of GLT8D2 in regulating macrophages are warranted and will provide additional insights.

## 5 Conclusions

Our study established a prognostic model based on glycosylation-related genes, which could contribute to assisting in clinical decision-making by predicting patient outcomes and recognizing responsiveness to particular therapies. Furthermore, increased expression of GLT8D2 is closely correlated with adverse prognosis and may underscore its role in regulating immune cell infiltration in GC patients, shedding new light on its potential key role as a prognostic biomarker related to immune infiltration in GC.

## Data availability statement

The datasets presented in this study can be found in online repositories. The names of the repository/repositories and accession number(s) can be found in the article/**Supplementary Material**.

## Ethics statement

This study was approved by the ethics committee of Guangdong Provincial People’s Hospital. The informed consent form signed by all patients was obtained from Shanghai Outdo Biotech Company. The studies were conducted in accordance with the local legislation and institutional requirements. Written informed consent for participation in this study was provided by the participants’ legal guardians/next of kin.

## Author contributions

HW: Writing – original draft, Data curation, Formal analysis, Investigation, Methodology, Project administration, Validation. JiZ: Writing – original draft, Data curation, Investigation, Methodology. QM: Data curation, Writing – original draft. JuZ: Funding acquisition, Resources, Supervision, Writing – review & editing. YL: Funding acquisition, Resources, Supervision, Writing – review & editing.
