# Supplementary material for: GLT8D2 is a prognostic biomarker and regulator of immune cell infiltration in gastric cancer
Source: Front Immunol. 2024 May 22;15:1370367. doi: 10.3389/fimmu.2024.1370367 (PMC11150579; doi:10.3389/fimmu.2024.1370367)
Supplement: Supplementary file 1 [file Table_1.docx]

**Table S1. The information of datasets from the GEO database**

| Accession number | Platform | Experiment type | Samples |
| --- | --- | --- | --- |
| GSE19826 | GLP570 | expression profiling by array | 27 |
| GSE54129 | GLP570 | expression profiling by array | 132 |
| GSE26899 | GLP6947 | expression profiling by array | 108 |
| GSE84433 | GLP6947 | expression profiling by array | 357 |
| GSE84437 | GLP6947 | expression profiling by array | 483 |
