# Supplementary material for: GLT8D2 is a prognostic biomarker and regulator of immune cell infiltration in gastric cancer
Source: Front Immunol. 2024 May 22;15:1370367. doi: 10.3389/fimmu.2024.1370367 (PMC11150579; doi:10.3389/fimmu.2024.1370367)
Supplement: Supplementary file 3 [file Table_3.docx]

https://www.jianguoyun.com/p/DRYFwbAQsrX5CRjBvLAFIAA
